# Dual observation of the ATP-evoked small GTPase activation and Ca^2+^ transient in astrocytes using a dark red fluorescent protein

**DOI:** 10.1038/srep39564

**Published:** 2016-12-22

**Authors:** Yoshihisa Nakahata, Junichi Nabekura, Hideji Murakoshi

**Affiliations:** 1 Division of Homeostatic Development, National Institute for Physiological Sciences, Okazaki, Aichi 444-8585, Japan; 2 Department of Physiological Sciences, The Graduate University for Advanced Studies (SOKENDAI), Okazaki, Aichi 444-8585, Japan; 3 Core Research for Evolutional Science and Technology, Japan Science and Technology Agency (JST), Kawaguchi, Saitama 332-0012, Japan; 4 Supportive Center for Brain Research, National Institute for Physiological Sciences, Okazaki, Aichi 444-8585, Japan; 5 Precursory Research for Embryonic Science and Technology, Japan Science and Technology Agency (JST), Kawaguchi, Saitama 332-0012, Japan

## Abstract

Intracellular signal transduction involves a number of biochemical reactions, which largely consist of protein-protein interactions and protein conformational changes. Monitoring Förster resonance energy transfer (FRET) by fluorescence lifetime imaging microscopy (FLIM), called FLIM-FRET, is one of the best ways to visualize such protein dynamics. Here, we attempted to apply dark red fluorescent proteins with significantly smaller quantum yields. Application of the dark mCherry mutants to single-molecule FRET sensors revealed that these dark mCherry mutants are a good acceptor in a pair with mRuby2. Because the FRET measurement between mRuby2 and dark mCherry requires only the red region of wavelengths, it facilitates dual observation with other signaling sensors such as genetically encoded Ca^2+^ sensors. Taking advantage of this approach, we attempted dual observation of Ca^2+^ and Rho GTPase (RhoA and Cdc42) activities in astrocytes and found that ATP triggers both RhoA and Cdc42 activation. In early phase, while Cdc42 activity is independent of Ca^2+^ transient evoked by ATP, RhoA activity is Ca^2+^ dependent. Moreover, the transient Ca^2+^ upregulation triggers long-lasting Cdc42 and RhoA activities, thereby converting short-term Ca^2+^ signaling to long-term signaling. Thus, the new FRET pair should be useful for dual observation of intracellular biochemical reactions.

Intracellular signal transduction involves a number of signaling proteins such as kinases^1^ and small GTPases^2^. These signaling molecules are spatiotemporally regulated for the cellular functions such as endocytosis, cytoskeletal reorganization, and gene expression in cells. One of the methods for research into such a signaling system in live cells is to monitor Förster resonance energy transfer (FRET), which allows us to detect protein-protein interactions and protein conformational changes^3^,^4^. For the detection of FRET, two-photon fluorescence lifetime imaging microscopy (2pFLIM) has been widely used in combination with fluorescent-protein-based FRET biosensors^5^,^6^. Because of good spectral separation and brightness of enhanced green fluorescent protein (EGFP), the pair consisting of EGFP as an energy donor and red fluorescent protein (RFP) as the acceptor is frequently used^5^,^7^,^8^. Nevertheless, this pair of fluorescent proteins occupies a wide range of wavelengths (500–650 nm) for the measurement, thus making it difficult to use additional fluorescent dyes or fluorescent proteins for dual observation.

A decade ago, a nonradiative yellow fluorescent protein called resonance energy-accepting chromoprotein (REACh) was developed and applied to 2pFLIM^9^. With EGFP as an energy donor and REACh as an energy acceptor, the molecular interaction between ubiquitin and its substrate were simultaneously visualized with RFP-labeled actin localization^9^. Recently, a maturation-improved REACh was also developed, and actin polymerization and spine volume change were simultaneously observed^10^. Furthermore, a dark green fluorescent protein called ShadowG has been developed and applied to dual observation of Ras FRET biosensor activity and mCherry-ERK translocation^11^. In addition to dark green/yellow fluorescent proteins, a red-shifted nonfluorescent protein named Ultramarine has been developed^12^. This protein has a broad red-shifted absorption spectrum (as compared to REACh) and an extremely low quantum yield (~0.001), potentially applicable to a wide range of fluorescent proteins for FRET measurement. Although various dark fluorescent proteins have already been developed, creation of a dark version of mCherry, which is a widely used red fluorescent protein^13^, has not been attempted. Here, we created two dark mCherry mutants by random mutagenesis and found that these mutants are good FLIM-FRET acceptors for a bright red fluorescent protein called mRuby2^14^. Because the pair “mRuby2 with dark mCherry” requires only narrow bandwidth (550–650 nm) for FLIM-FRET measurements, we employed this pair with a green fluorescent protein-based calcium sensor G-GECO, which uses the bandwidth (500–550 nm) for dual observation of Ca^2+^ and Rho GTPase activity in astrocytes.

Astrocytes are the most abundant glial cell type in the central nervous system and play essential roles in maintaining the structure, metabolism, and synaptic functions of the neural network^15^,^16^. It is widely accepted that astrocytes are activated by extracellular signals such as adenosine 5′-triphosphate (ATP) and glutamate^17^. Nonetheless, intracellular signaling cascades besides calcium have largely remained elusive. Here, using a newly developed FRET pair, we monitored the temporal activity pattern of Ca^2+^ and the activities of cell division cycle 42 (Cdc42) or Ras homolog A (RhoA). These are members of the Rho family of small GTPases and are known to play pivotal roles in morphological changes and migration of cells by regulating actin polymerization^2^. We found that extracellular ATP triggers robust Cdc42 and RhoA activation with the early/transient phase and late/sustained phase. In the early phase, although Cdc42 activity is independent of the Ca^2+^ transient evoked by ATP, RhoA activity is dependent on Ca^2+^ transient. Moreover, the late-phase activities of both Cdc42 and RhoA are triggered by transient Ca^2+^ upregulation, converting the short-term signaling to the long-term signaling. Thus, an ATP-evoked calcium transient temporally regulates Cdc42 and RhoA activities in astrocytes.

## Results

**Figure 1:**
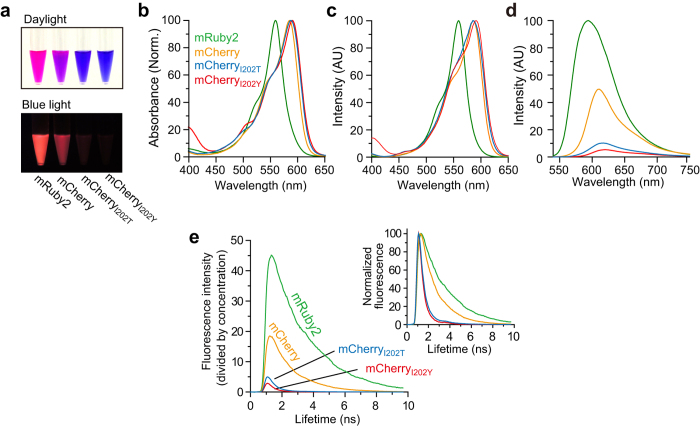
Spectrofluorimetric analysis of purified dark mCherry mutants. **(a)** Purified fluorescent proteins (300 μM) in daylight (top) and under blue light-emitting diode (LED) light (bottom). **(b**,**c)** Normalized absorption (**b**) and excitation (**c**) spectra of mRuby2 and mCherry mutants. **(d)** The emission spectra of mRuby2, mCherry, and mCherry mutants excited at 525 nm. For each sample, optical density at 525 nm was adjusted to 0.15. **(e)** The fluorescence lifetime curves of the fluorescent proteins subjected to 1000-nm two-photon excitation.

To create a dark red fluorescent protein, we carried out error-prone PCR-based random mutagenesis. We introduced the mutations into mCherry between amino acid positions L118 and K236, avoiding introduction of any mutations into the chromophore. After construction of a genetic library, we screened it for dark mCherry mutants that have a high extinction coefficient but a low quantum yield among 100,000 colonies, and identified colonies with a vivid color in daylight. This vivid color is most likely due to strong absorption of light by the fluorescent protein in these bacteria. Subsequently, we further examined the identified colonies under blue light to screen the dark colonies in a way similar to a previously reported method^11^,^18^. As a result, we identified two colonies that had a blue color in daylight but showed dark fluorescence under blue light. The purified mutants are also blue in daylight and dark under blue light (Fig. 1a). The sequence analysis identified two mutants that have the mutation I202Y or I202T (i.e., mCherry_I202Y_ and mCherry_I202T_). These mutations are the same as the previously reported mutations yielding longer-emission mutants^19^,^20^. Consistent with the previous data^20^, our spectral analysis confirmed that the absorption, excitation, and emission spectra are slightly shifted to a longer wavelength as compared with those of mCherry (Fig. 1b–d and Table 1). Further saturated mutagenesis at the position of 201, 202, and 203 did not yield mCherry mutants which has better absorption and darkness. Extinction coefficients of mCherry_I202Y_ and mCherry_I202T_ are half that of mCherry (Table 1), and quantum yields are 0.02 and 0.04, which are ~8- and 4-fold smaller than the quantum yield of mCherry, respectively (Table 1). In line with these results, the fluorescence lifetime values of purified mCherry_I202Y_ (0.52 ns) and mCherry_I202T_ (0.60 ns) are much shorter than that of mCherry (Fig. 1e, Table 1). Similar results were obtained in live HeLa cells (mCherry: 1.54 ns, mCherry_I202Y_: 0.45, mCherry_I202T_: 0.52; Table 1). In our optical setup, the photon contamination calculated by comparing the areas of lifetime curves of mRuby2 and dark mCherrys are 1.7 and 3.4% for mCherry_I202Y_ and mCherry_I202T_, respectively (Fig. 1e).

**Figure 2:**
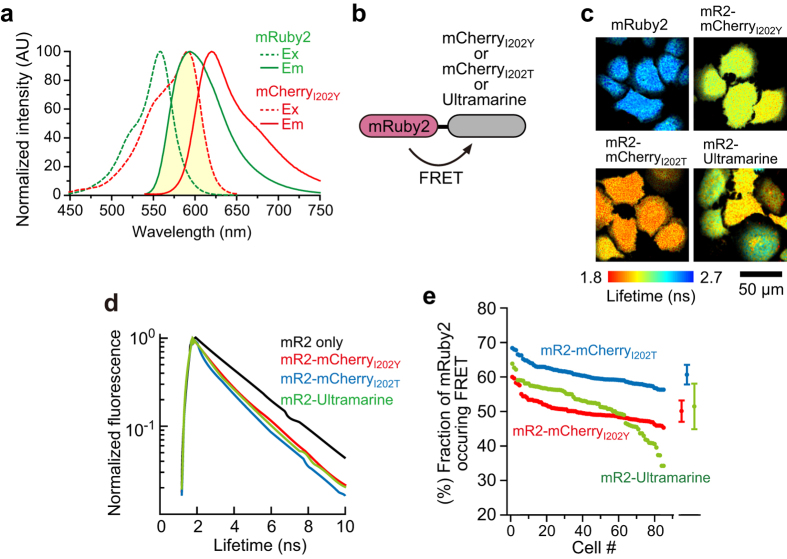
FRET efficiency and the proportion of matured mCherry mutants. **(a)** The spectral overlap (yellow region) between the mRuby2 emission spectrum and mCherry_I202Y_ excitation spectrum. AU: arbitrary units. **(b)** A schematic drawing of the tandem fluorescent protein used for evaluation of the FRET and chromophore maturation efficiency. **(c)** Representative fluorescence lifetime images of the mRuby2 and mRuby2-based tandem fluorescent proteins in HeLa cells; the images were taken at 1000-nm two-photon excitation. Because the expression level of mRuby2-Ultramarine was low, laser power of 2 mW was used, while 1 mW was used for mRuby2, mRuby2-mCherry_I202Y_, and mCherry_I202T_. The scale bar is 50 μm. **(d)** Lifetime curves of the mRuby2-based tandem fluorescent proteins in HeLa cells. **(e)** Fraction of mRuby2 occuring FRET of mRuby2 and mRuby2-based tandem fluorescent proteins in individual cells plotted in the descending order. The data plots are also presented as mean ± SD on the right (p < 0.05, analysis of variance [ANOVA] followed by Scheffé’s *post hoc* test).

Next, we aimed to utilize these mCherry mutants as an acceptor for FLIM-FRET measurement, and we chose mRuby2^14^ as the energy donor of FRET. An advantage of mRuby2 is that because it is a red fluorescent protein, the pair “mRuby2 with dark mCherrys” occupies only the red band (550–700 nm) for FRET measurement, thereby facilitating dual observation with green fluorescent dyes. Because the emission spectrum of mRuby2 significantly overlaps with the excitation spectra of mCherry mutants (Fig. 2a), FRET should take place within these pairs in a distance-dependent and orientation-dependent manner. To test this idea, we expressed a tandem construct consisting of mRuby2 and dark mCherry in HeLa cells (Fig. 2b) and measured the fluorescence lifetime of mRuby2 using 2pFLIM (Fig. 2c,d). When the fluorescence lifetime of mRuby2-mCherry_I202Y_ or mRuby2-mCherry_I202T_ tandems was measured (2.11 ns and 2.00 ns, respectively), these values turned out to be much smaller than the lifetime of mRuby2 (2.52 ns) (Fig. 2d). These results indicated that the FRET from mRuby2 to dark mCherry mutants was taking place. Furthermore, we paired Ultramarine, which is a known dark fluorescent protein^12^, with mRuby2, and found that FRET also occurs in the mRuby2-Ultramarine pair (Fig. 2c,d). Next, we quantified the cell-to-cell variability of the proportion of mRuby2 undergoing FRET as described previously (Fig. 2e)^11^,^21^. The cell-to-cell variability was advantageously lower for the mRuby2-mCherry_I202Y_/mCherry_I202T_ pair compared with mRuby2-Ultramarine in our experimental condition, suggesting that the dark mCherry mutants are more stable FRET acceptors than Ultramarine is when paired with mRuby2.

To test the performance of the dark mCherry mutants extensively, we compared the two single-molecule types of the CaMKII FRET sensors (Camuiα) differing in their acceptor fluorophores (Fig. S1). mCherry _I202Y_ or mCherry_I202T_ was fused to the C terminus of mRuby2-CaMKIIα, creating Camuiα-mRmC_I202Y_ or Camuiα-mRmC_I202T_: a configuration similar to previously reported formats^21^,^22^,^23^. To compare the performance of these sensors, we transfected HeLa cells with Camuiα-mRmC_I202Y_ or Camuiα-mRmC_I202T_, and compared the response signals by 2pFLIM^5^,^21^,^23^,^24^. Because Ca^2+^ activates calmodulin, and subsequent binding to CaMKII induces a structural change of CaMKII (Fig. S1a)^25^, we used an ionophore for inducing Ca^2+^ influx into cells and for activation of CaMKII. Stimulation with the ionophore rapidly activated CaMKII, and the signal reached a plateau within a few minutes (Fig. S1b–e). A comparison of FRET signals revealed that Camuiα-mRmC_I202Y_ and Camuiα-mRmC_I202T_ have similar response signals (Fig. S1c–g). Next, we compared the performance of dark mCherry mutants using a light-sensitive LOV2-Jα helix domain from phototropin 1, which absorbs blue light and reversibly transforms its conformation from a closed to open state (Fig. S2)^26^,^27^. mRuby2 and mCherry_I202Y_ or mCherry_I202T_ were fused to the N and C termini of LOV2, respectively, creating LOV2-mRmC_I202Y_ and LOV2-mRmC_I202T_ (Fig. S2a). To evaluate the performance of dark mCherry mutants, we transfected HeLa cells with LOV2 sensors and monitored the blue-light-dependent structural changes by means of 2pFLIM (Fig. S2b–g). We found that the response signal of LOV2-mRmC_I202Y_ was slightly better than that of LOV2-mRmC_I202T_ (Fig. S2c).

**Figure 3:**
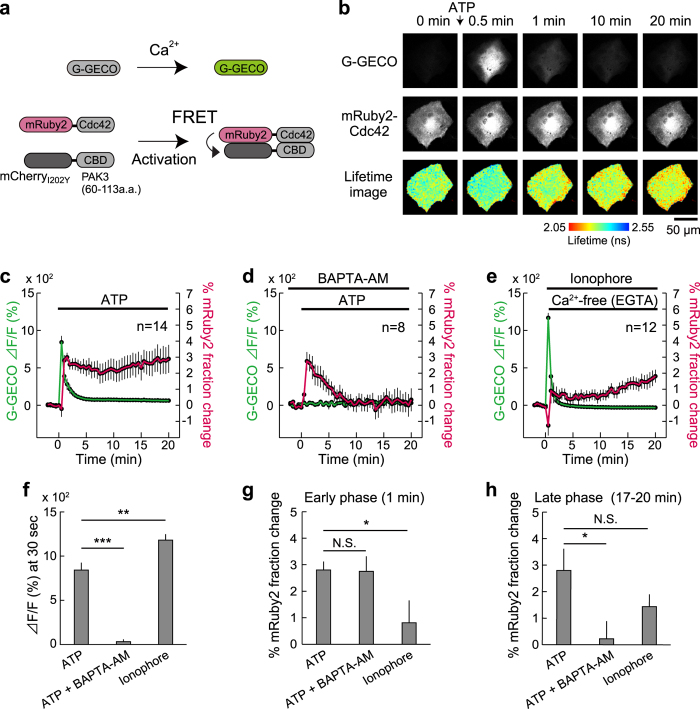
Dual observation of a Ca^2+^ transient and Cdc42 activation in astrocytes. **(a)** A schematic of the G-GECO1.1 and Cdc42 FRET sensor activation. mCherry_I202Y_-CBD and mRuby2-Cdc42 are fused via P2A sequence (mCherry_I202Y_-CBD-P2A-mRuby2-Cdc42), leading to the equal amount of expression in cells. **(b)** Representative time-lapse images of dual observation of G-GECO fluorescence ([Ca^2+^] increase) (top), the fluorescence of mRuby2-Cdc42 (middle), and their fluorescence lifetime (bottom) in a cultured astrocyte after stimulation with 100 μM ATP. The scale bar is 50 μm. **(c**–**e)** Time course analyses of the Ca^2+^ response (green) and Cdc42 activation (the change in the mRuby2-Cdc42 proportion [fraction] undergoing FRET; magenta) after the application of ATP (**c**; n = 14), ATP application during Ca^2+^ chelation by means of 200 μM BAPTA-AM (**d**; n = 8), and application of 10 μM ionophore (**e**; n = 12). For the ionophore experiment, a nominal Ca^2+^-free buffer was used 30 s after ionophore application to stop the Ca^2+^ influx. **(f–h)** Quantitative analysis of the Ca^2+^ transient and Cdc42 activation. G-GECO fluorescence intensity at 30 s (**f**), the fraction of mRuby2-Cdc42 undergoing FRET (**g**,**h**) at the indicated time points after the respective stimulation data were compared. The data are presented as mean ± SEM. Statistical significance was determined by one-way analysis of variance (ANOVA) followed by Dunnett’s test. *P < 0.05, **P < 0.01, ***P < 0.001, N.S.: not significant.

**Figure 4:**
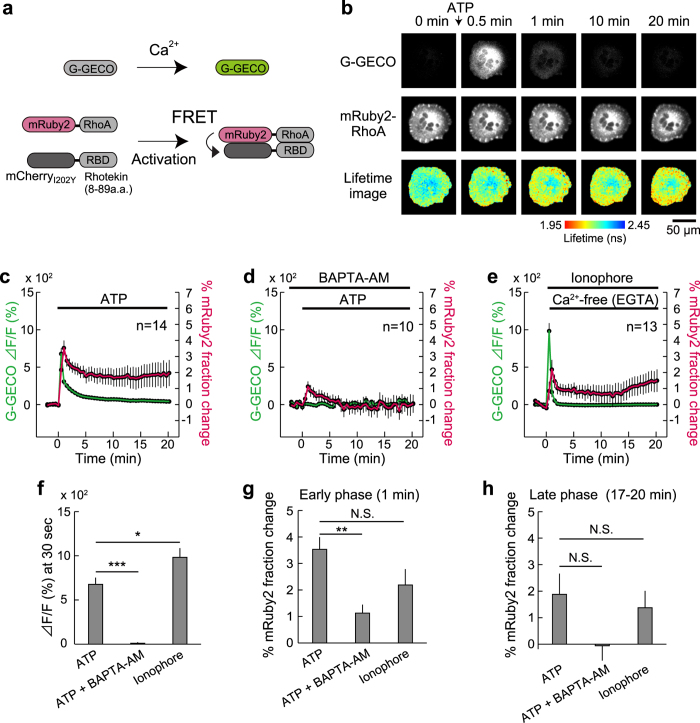
Dual observation of a Ca^2+^ concentration increase and RhoA activation in astrocytes. **(a)** A schematic of the G-GECO1.1 and RhoA FRET sensor activation. mCherry_I202Y_-RBD and mRuby2-RhoA are fused via P2A sequence (mCherry_I202Y_-RBD-P2A-mRuby2-RhoA), leading to the equal amount of expression in cells. **(b)** Representative time-lapse images of dual observation of G-GECO fluorescence ([Ca^2+^] increase) (top), the fluorescence of mRuby2-RhoA (middle), and their fluorescence lifetime (bottom) in a cultured astrocyte after stimulation with 100 μM ATP. The scale bar is 50 μm. **(c–e)** Time course data on the Ca^2+^ response and RhoA activation (the change in the mRuby2-RhoA proportion [fraction] undergoing FRET; magenta) after ATP application (**f**; n = 14), ATP application during Ca^2+^ chelation by means of 200 μM BAPTA-AM (**d**; n = 8), and application of 10 μM ionophore (**e**; n = 12). For the ionophore experiment, a nominal Ca^2+^-free buffer was applied 30 s after ionophore application to stop Ca^2+^ influx. **(f–h)** Quantitative analysis of the calcium transient and RhoA activation. G-GECO fluorescence intensity at 30 s (**f**) and the fraction of mRuby2-RhoA undergoing FRET (**g**,**h**) were compared at the indicated time points after respective stimulation. The data are presented as mean ± SEM. Statistical significance was evaluated by one-way ANOVA followed by Dunnett’s test. *P < 0.05, **P < 0.01, ***P < 0.001, N.S., not significant.

Because the pair of mRuby2 and dark mCherry requires only a narrow range of wavelengths (550–650 nm) for observation (Fig. 2a), we next attempted dual observation with a GFP-based calcium indicator in astrocytes. It has been shown that ATP induces a cytosolic Ca^2+^ transient in astrocytes^28^,^29^,^30^, and this process may lead to the release of gliotransmitters such as ATP and glutamate for modulation of neuronal activity^15^,^17^. In spite of the long history of calcium research, temporal activity patterns of Rho GTPases and their involvement in Ca^2+^-related processes have remained poorly studied. To monitor the activity of Cdc42 and RhoA, we developed FRET sensors based on the mRuby2-and-mCherry_I202Y_ pair using a previously reported strategy (Figs 3a and 4a)^24^. We chose mCherry_I202Y_ rather than mCherry_I202T_ because of the slightly wider dynamic range (Figs S1c and S2c). For the Cdc42 FRET sensor, mRuby2 was fused to Cdc42 as an energy donor, and mCherry_I202Y_ was fused to an activated Cdc42 binding domain (CBD) derived from PAK3 as an acceptor, as described previously^24^. When mRuby2-Cdc42 was activated, mCherry_I202Y_-CBD bound to the activated mRuby2-Cdc42, leading to FRET between mRuby2 and mCherry_I202Y_ (Fig. 3a). For a RhoA FRET sensor, a similar strategy was used with an activated RhoA-binding domain derived from Rhotekin as an acceptor (Fig. 4a)^24^. We electroporated the Rho GTPase FRET sensor and a genetically encoded calcium indicator, G-GECO1.1^31^ into cultured astrocytes and monitored Rho GTPase activity and Ca^2+^ concentration simultaneously.

To test whether extracellular ATP triggers Cdc42/RhoA activation, we used a saturable concentration of ATP (100 μM) with astrocytes expressing the FRET sensor, and simultaneously monitored the GTPase activity and Ca^2+^ changes by means of 2pFLIM. After ATP stimulation, a cytosolic Ca^2+^ transient occurred within 30 s (Figs 3b,c and 4b,c), most likely via P2 receptors^32^, in agreement with other reports^28^,^29^,^30^. Delayed by the calcium transient, both Cdc42 and RhoA were activated within 1 min, and the activities persisted for >20 min (Figs 3c and 4c). Next, we tested whether Cdc42/RhoA activation depends on the Ca^2+^ upregulation evoked by ATP. Cells were stimulated with ATP in the presence of a cell-permeable calcium chelator (200 μM BAPTA-AM) in combination with removal of extracellular Ca^2+^. Imaging of Ca^2+^ confirmed strong inhibition of the ATP-evoked cytosolic Ca^2+^ upregulation (Figs 3d and f, 4d and f). It is noteworthy that in the early phase (1 min after ATP simulation), although Cdc42 activation is Ca^2+^ independent (Fig. 3g), RhoA activation is Ca^2+^ dependent (Fig. 4g), suggesting that Cdc42 and RhoA are activated differently by ATP. In contrast to the early-phase activities, the late-phase activities of both Cdc42 and RhoA were strongly reduced by Ca^2+^-inhibition (Figs 3g and 4g), implying that the early and late phases are regulated independently. To confirm the calcium dependence more directly, the cytosolic Ca^2+^ upregulation was triggered by a selective calcium ionophore (Figs 3e,f and 4e,f). We bath-applied 10 μM ionophore to induce Ca^2+^ influx into the cytosol from the extracellular pool and subsequently added a sufficient amount of a calcium chelator (10 mM EGTA in calcium-free buffer) to stop the Ca^2+^ influx. This protocol effectively mimics the temporal pattern of the Ca^2+^ transient evoked by ATP (Figs 3e and 4e). The direct increase in cytosolic Ca^2+^ concentration does not activate Cdc42 in the early phase (Fig. 3e,g) but gradually activates it during 20 min, suggesting that the late-phase activity of Cdc42 is Ca^2+^ dependent (Fig. 3e,h). In contrast to Cdc42, the Ca^2+^ increase directly activated RhoA in early and late phases (Fig. 4e,g,h), suggesting that RhoA activity is Ca^2+^ dependent. As negative control experiments, we used Rho GTPase deficient FRET sensors, and confirmed that the binding fraction of mRuby2 does not increase after ATP stimulation (Fig. S3). Furthermore, by taking advantage of dual observation, we compared the Ca^2+^ transient and Rho GTPase activity in early/late phases in individual cells (Fig. S4), and found that the significant correlation between ionophore-evoked Ca^2+^ transient and RhoA activity at early phase (Fig. S4c), implying that the profound relationship between the level of Ca^2+^ and RhoA activity.

## Discussion

Here, we successfully demonstrated that the pair “mRuby2 and a dark red fluorescent protein” is suitable for FLIM-FRET measurement (Figs 2 and S1 and S2). Notably, because such pairs require only a narrow range of wavelengths (550–650 nm), they facilitate dual observation with a green fluorescent dye or protein using the 500–550 nm range of wavelengths (Figs 3 and 4). Although chromoprotein named Ultramarine was reported as an acceptor for FLIM-FRET, and its spectral properties are similar to those of dark mCherrys^12^, the reduced cell-to-cell variability of dark mCherrys represents a big advantage over Ultramarine (Fig. 2e).

**Figure 5:**
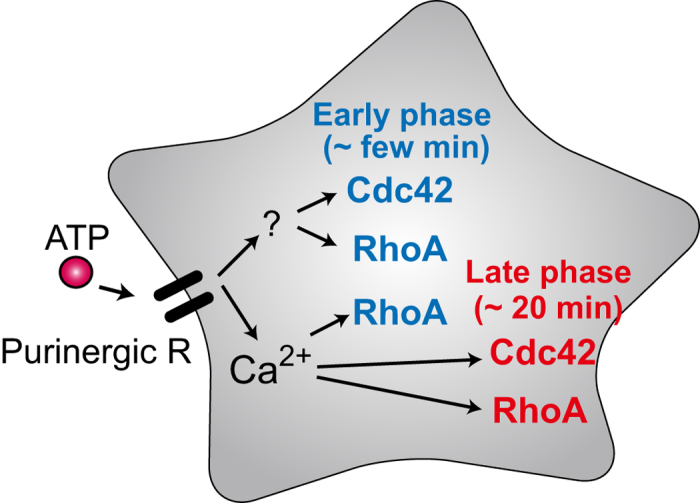
A schematic of the relations between Cdc42/RhoA activities and the Ca^2+^ transient in astrocytes. Extracellular ATP presumably activates purinergic receptors on the plasma membrane, leading to activation of GTPases (Cdc42/RhoA). Although early-phase activity of Cdc42 is Ca^2+^ independent, RhoA activity is regulated by both Ca^2+^-dependent and Ca^2+^-independent pathways. In contrast to the differential Cdc42/RhoA activation in the early phase, late-phase activities of both Cdc42 and RhoA depend on the Ca^2+^ transient.

To demonstrate that the pair mRuby2 and dark mCherry is a powerful tool for research on intracellular signal transduction mechanisms, we utilized this pair to dissect signaling mediated by small GTPases and their relations with Ca^2+^. We found that in early phase, while Cdc42 activity is independent of Ca^2+^ transient evoked by ATP, RhoA activity is Ca^2+^ dependent (Figs 3 and 4). Moreover, the transient Ca^2+^ upregulation triggers long-lasting Cdc42 and RhoA activities, thereby converting short-term signaling to long-term signaling (Fig. 5). Although the function of these long-lasting activities has yet to be determined, various studies suggest that Cdc42 and RhoA activities contribute to the various functions such as cell migration, formation of actin stress fibers, and inhibition of the stellation^33^,^34^,^35^,^36^,^37^. These findings may represent key information for future experiments.

Taken together, our data show that dark mCherry can be paired with mRuby2 to serve as FRET probes and may substantially facilitate dual observation of biochemical signaling events.

## Materials and Methods

### Random mutagenesis

Random mutagenesis was performed by amplifying *mCherry* (the fragment corresponding to amino acid positions 118–236) by error-prone PCR using the Diversity PCR Random Mutagenesis Kit with a high error rate (7.2 per 1 kbp; Takara). Subsequently, the PCR fragments were digested with *Pst*I and *BsrG*I and ligated into the mCherry-pRSET vector. *Escherichia coli* DH5α cells were transformed with the plasmid library by electroporation and grown for 18–20 h at 34 °C on Luria-Bertani (LB) agar plates supplemented with antibiotics.

### Plasmid construction

CMV-G-GECO1.1 was a gift from Robert Campbell (Addgene plasmid # 32445). For construction of the mRuby2 plasmid, the *mRuby2* gene was amplified by PCR from the pcDNA3-AKAR2-CR plasmid (a gift from Michael Lin, Addgene plasmid # 40255) as a template and was inserted into the modified pEGFP-C1 vector by replacing *EGFP*. This vector was used in all mammalian-expression experiments in this study. For construction of tandem fluorescent protein plasmids, gene *mCherry*_*I202Y*_ or *mCherry*_*I202T*_ or the synthesized *Ultramarine* gene was inserted into the multiple cloning site in the *FLAG*-tagged *mRuby2* plasmid (encoding amino acid residues 1–229 of mRuby2) with a linker encoding the peptide SRAQASNSAVDGTAGPGSG. For construction of mRuby2-based CaMKIIα FRET sensors, mRuby2-CaMKIIα with the linker peptide SGLRSRA was fused to *mCherry*_*I202Y*_, *mCherry*_*I202T*_, or *Ultramarine* with a linker peptide GSG. For construction of the mRuby2-based LOV2 FRET sensors, we fused *mRuby2* (DNA sequence corresponding to amino acid residues 1–234) to the N terminus of the LOV2 domain (DNA sequence corresponding to amino acid residues 404–546 in phototropin 1) with a linker encoding the peptide ASM. Then, *mCherry*_*I202Y*_, *mCherry*_*I202T*_, or *Ultramarine* was subcloned into the C-terminal region of LOV2 with the linker peptide KLGNS. For construction of the RhoA FRET sensor, we fused *mCherry*_*I202Y*_ to the binding domain of Rhotekin (amino acid residues 8–89) with the linker peptide SGLRS. Subsequently, mRuby2-RhoA with the linker peptide SGLRSRG was fused to the C terminus of the above protein via the P2A sequence^38^ so that the RhoA-binding domain and RhoA parts were translated into different polypeptides within the cell. By means of this construct, the Cdc42 FRET sensor was created via replacement of the binding domain of Rhotekin and RhoA with the binding domain of PAK3 (amino acid residues 60–113) and Cdc42, respectively.

### Properties of the fluorescent proteins

For purification of His-tagged proteins, *mRuby2, mCherry, mCherry*_*I202Y*_, or *mCherry*_*I202T*_ gene was inserted into the pRSET vector (Invitrogen). The respective proteins were overexpressed in *E. coli* DH5α cells and purified on a Ni^+^-nitrilotriacetate column (HiTrap, GE Healthcare) with subsequent dialysis against PBS. Absorption, excitation, and emission spectra of the fluorescent proteins were recorded on a UV-Vis spectrophotometer (UV-1800; Shimadzu) or a fluorescence spectrophotometer (RF-6000; Shimadzu). The molar concentration of the purified proteins was measured by the Bradford protein assay (Bio-Rad). The molar extinction coefficients were determined by dividing the peak optical density by the molar concentration determined by the alkaline denaturation method^20^,^39^,^40^. The quantum yields of the proteins were determined as described elsewhere^41^. First, optical densities of the fluorescent proteins in PBS were adjusted to 0.15. Using the fluorescence spectrophotometer, the area of spectral traces of fluorescence emission, obtained at 525-nm excitation, was integrated over 540–750 nm and divided by the area for the Rhodamine 101 trace, then multiplied by the quantum yield (1.0) of Rhodamine 101 diluted in ethanol^42^.

### HeLa cell culture and transfection

HeLa cells were cultured in the Dulbecco’s modified Eagle’s medium (DMEM) (supplemented with 10% of fetal bovine serum) at 37 °C in a humidified atmosphere containing 5% of CO_2_. Then, the cells were transfected with the plasmids using Lipofectamine 3000 (Invitrogen), followed by incubation for 18–24 h. FLIM-FRET imaging was conducted in a solution containing 4-(2-hydroxyethyl)-1-piperazineethanesulfonic acid (HEPES; 30 mM, pH 7.3)-buffered artificial cerebrospinal fluid (130 mM NaCl, 2.5 mM KCl, 1 mM CaCl_2_, 1 mM MgCl_2_, 1.25 mM NaH_2_PO_4_, and 25 mM glucose) at room temperature.

### Astrocyte culture and transfection

All animal procedures were approved by the National Institutes of Natural Sciences Animal Care and Use Committee and were performed in accordance with the relevant guidelines and regulations. Astrocytic culture was prepared from cerebral cortices of newborn (P0) C57BL/6 J mice (SLC, Hamamatsu, Japan) after deep anesthesia and decapitation. The tissues were dissociated in cold Hank’s Balanced Saline Solution (HBSS, Invitrogen) by trituration as described previously^43^. The astrocytes were maintained in the culture medium (DMEM; Sigma-Aldrich, St. Louis, MO, USA) supplemented with 10% of fetal bovine serum (Sigma), 5 μg/mL bovine insulin (Sigma-Aldrich), and 0.2% glucose in a 75-cm^2^ culture flask (250 ml, BD falcon). The culture medium was exchanged with the plating medium after 2 days *in vitro* and once a week. When the cultured cells reached confluence at 10–14 days *in vitro*, the culture flask was rinsed twice with HBSS as a conventional shake-off protocol to remove nonadherent cells. Adherent cells were trypsinized, and cDNA was transfected into these cells by means of an Amaxa Nucleofector (Lonza, program U-023). Transfected astrocytes were plated at the density of 1.2 × 10^6^ cells/dish in 35-mm culture dishes. FLIM-FRET imaging was carried out in solution (10 mM HEPES, 148 mM NaCl, 5 mM KCl, 2 mM CaCl_2_, 1 mM MgCl_2_, and 10 mM glucose, pH 7.4) at room temperature. BAPTA were used in a Ca^2+^-free solution (10 mM HEPES, 10 mM EGTA, 148 mM NaCl, 5 mM KCl, 3 mM MgCl_2_, and 10 mM glucose, pH 7.4).

### Two-photon fluorescence lifetime imaging

A custom-made two-photon fluorescence lifetime imaging microscope was used for this analysis as described elsewhere^5^. Briefly, mRuby2 in the FRET sensor was excited with a Ti-sapphire laser (Mai Tai; Spectra-Physics) tuned to 1000 nm. The X and Y scanning mirrors (6210 H; Cambridge Technology) were controlled with a PCI board (PCI-6110; National Instruments) and ScanImage software^44^. The photon signals of mRuby2 fluorescence were collected by means of an objective lens (60×, 0.9 NA; Olympus) and a photomultiplier tube (H7422–40p; Hamamatsu) placed after a dichroic mirror (FF553-SDi01; Semrock) and emission filter (FF01-625/90; Semrock). For dual observation, the FF02-641/75 emission filter (Semrock) was used instead. For Ca^2+^ imaging, G-GECO1.1 fluorescence was detected by means of a photomultiplier tube (R3896; Hamamatsu) placed after the emission filter (FF01-510/84; Semrock). A fluorescence lifetime curve was recorded by means of a time-correlated single-photon-counting board (SPC-150; Becker & Hickl) controlled with custom software^5^. For construction of a fluorescence lifetime image, the mean fluorescence lifetime values (τ_m_) in each pixel were translated into a color-coded image^45^. Analysis of the fluorescence lifetime change was carried out as described previously^46^. For stimulation, an ionophore (4-Bromo-A23187) was purchased from Funakoshi Co., Ltd. ATP and BAPTA-AM were purchased from Sigma-Aldrich.

### Quantification of the proportion of mRuby2 undergoing FRET

We quantified free mRuby2 and mRuby2 undergoing FRET as described elsewhere^5^. Briefly, the fluorescence lifetime curve was fitted to a double-exponential function convolved with an instrument response function. In case of the tandem fluorescent protein, we assumed that two fractions exist in the cells: 1) mature mRuby2 fused to an immature acceptor fluorescent protein where fluorescence lifetime of mRuby2 (τ_free_) is 2.52 ns, the same as that of free mRuby2; 2) mature mRuby2 fused to a mature acceptor fluorescent protein where FRET takes place and fluorescence lifetime of mRuby2 (τ_FRET_) gets shorter. To measure τ_FRET_, we used the following formula as described previously^46^:


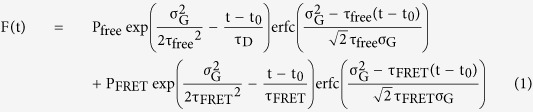


In Eq. (1), erfc is a complementary error function, t_0_ is the time offset, σ_G_ is the standard deviation of the IRF, and τ_free_ and τ_FRET_ are the decay time constants of free mRuby2 and mRuby2 with FRET, respectively. P_free_ and P_FRET_ are the coefficients of the free mRuby2 component and mRuby2 with FRET, respectively. Using this formula, we obtained τ_FRET_ for mRuby2-mCherry_I202Y_ (0.80 ns), mRuby2-mCherry_I202T_ (0.74 ns), and mRuby2-Ultramarine (0.84 ns). Using the obtained τ_free_ and τ_FRET_, we calculated the proportion of mRuby2 undergoing FRET in individual cells using the following formula as described elsewhere^5^,^46^:


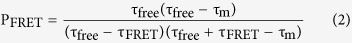


## Additional Information

**How to cite this article**: Nakahata, Y. *et al*. Dual observation of the ATP-evoked small GTPase activation and Ca^2+^ transient in astrocytes using a dark red fluorescent protein. *Sci. Rep.* **6**, 39564; doi: 10.1038/srep39564 (2016).

**Publisher's note:** Springer Nature remains neutral with regard to jurisdictional claims in published maps and institutional affiliations.

## Supplementary Material

Supplementary Information

## Figures and Tables

**Table 1:** The characteristics of fluorescent proteins used in the study.

Protein	EC (M^−1^cm^−1^)	QY	Abs (nm)	Ex (nm)	Em (nm)	Relative brightness	Förster distance from mRuby2 (nm)	Fluorescence lifetime of purified proteins (ns)	Fluorescence lifetime in cells (ns)
mRuby2	113,000*	0.38*		559*	600*	100	—	2.58	2.52
mCherry	78,000	0.17	585	585	611	31	—	1.40 (79%), 2.90 (21%)	1.54
	72000†	0.22†	—	—	—	—	—		
mCherry (I202Y)	32,000	0.02	590	592	620	2	4.9	0.52	0.45
	79,000‡	0.03‡	—	—	—	—	—	—	—
mCherry (I202T)	42,000	0.04	587	587	617	4	5.1	0.60	0.52
	79,000‡	0.05‡	—	—	—	—	—	—	—

Extinction coefficients were measured by the alkaline denaturation method (see *Methods*). The quantum yields were determined using Rhodamine 101 in EtOH as a reference (see *Methods*). Abs: absorption maximum, E_x_: excitation maximum, E_m_: emission maximum. ^*,†,‡^Data from other studies^13^,^14^,^20^. For purified mCherry, because the single exponential does not fit the fluorescence lifetime curves, we fitted the curves to a double exponential function convolved with an instrument response function^5^. Since the measurement of EC and QY could be operation sensitive, we carried out the side-by-side measurement of mCherry as a control and confirmed that the values are comparable with the previous report^13^.
