# Supplementary material for: Dual observation of the ATP-evoked small GTPase activation and Ca2+ transient in astrocytes using a dark red fluorescent protein
Source: Sci Rep. 2016 Dec 22;6:39564. doi: 10.1038/srep39564 (PMC5177924; doi:10.1038/srep39564)
Supplement: Supplementary Information [file srep39564-s1.pdf]

Supplementary information

Dual observation of the ATP-evoked small GTPase activation and Ca^{2+} transient in astrocytes using a dark red fluorescent protein

Yoshihisa Nakahata[1], Junichi Nabekura[1,2,3], and Hideji Murakoshi[2,4,5,]*

[1] Division of Homeostatic Development, National Institute for Physiological Sciences, Okazaki, Aichi 444-8585, Japan, [2] Department of Physiological Sciences, The Graduate University for Advanced Studies (SOKENDAI), Okazaki, Aichi 444-8585, Japan, [3] Core Research for Evolutional Science and Technology, Japan Science and Technology Agency (JST), Kawaguchi, Saitama 332-0012, Japan, [4] Supportive Center for Brain Research, National Institute for Physiological Sciences, Okazaki, Aichi 444-8585, Japan, [5] Precursory Research for Embryonic Science and Technology, Japan Science and Technology Agency (JST), Kawaguchi, Saitama 332-0012, Japan.

*Correspondence author: Hideji Murakoshi; e-mail: murakosh@nips.ac.jp; Tel.: +81 564-55-7857; Fax: +81 564-55-7858

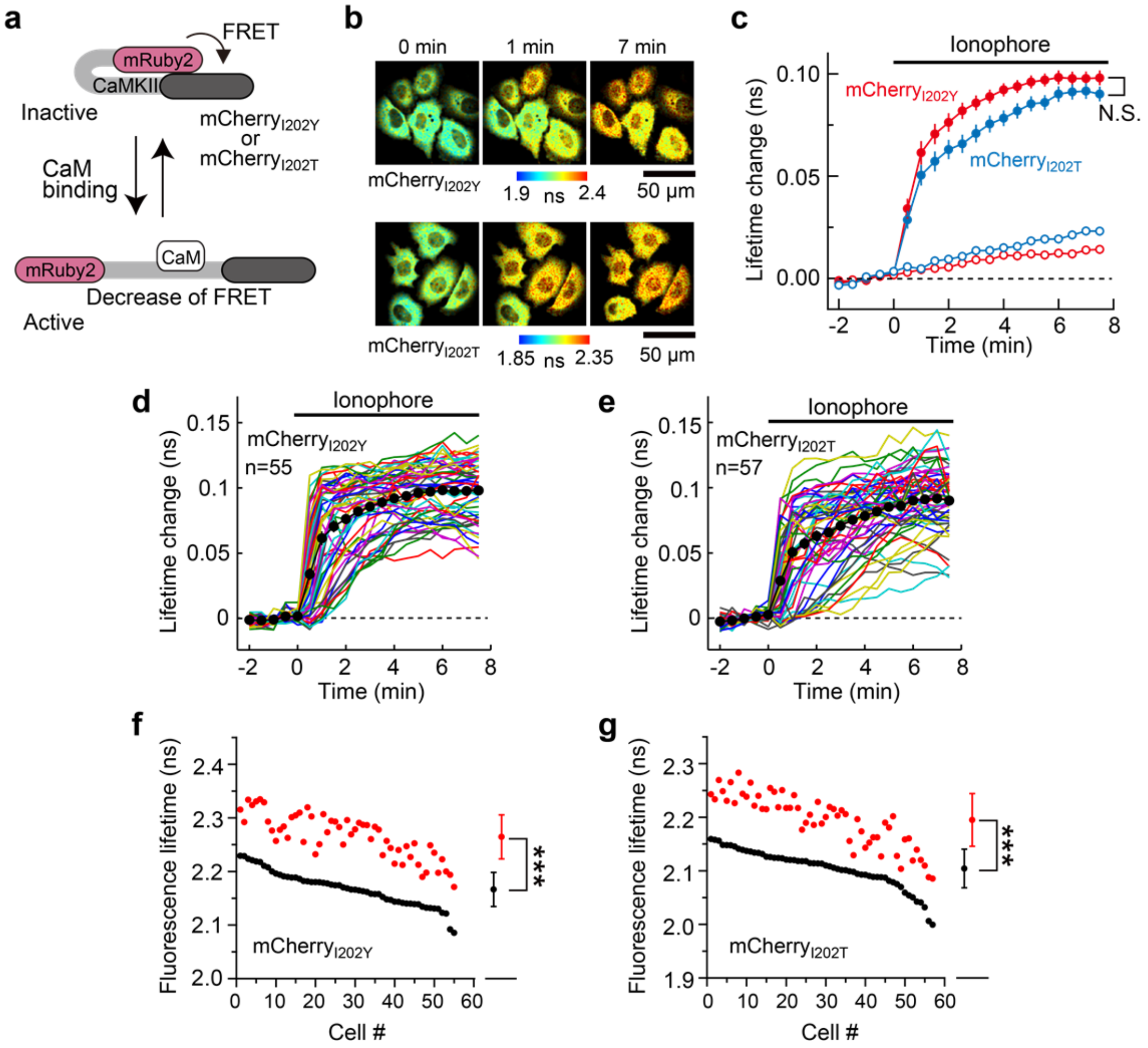

Figure S1. Performance of mCherry mutants in Camuiα.

(a) A schematic drawing of activation of the Camuiα-mRmC mutant.

(b) Representative fluorescence lifetime images of Camuiα FRET sensors coexpressed with calmodulin (DNA molar ratio 1:1) in HeLa cells after stimulation with 10 µM 4-Bromo-calcium ionophore (A23187). Two-photon excitation at 1000 nm was used for excitation of mRuby2. The scale bar is 50 µm.

(c) Fluorescence lifetime changes in response to application of the ionophore (filled circles) or dimethyl sulfoxide (DMSO; open circles). The number of cells analyzed is 55 for mCherry$_{I202Y}$ and 57 for mCherry$_{I202T}$. In the DMSO experiment, the number of cells analyzed

is 35 for $mCherry_{I202Y}$ and 38 for $mCherry_{I202T}$. The data are presented as mean ± SEM. Asterisks denote statistical significance (*t* test; $p < 0.001$).

(d, e) Fluorescence lifetime changes of Camuiα sensors in individual cells after stimulation with an ionophore (the same dataset as in panel c). Colored lines represent the response signals from individual cells, and the black circles indicate an averaged time course. The data are presented as mean ± SEM.

(f, g) The basal fluorescence lifetime (averaged over −2 to 0 min) of individual cells is plotted in the descending order (black) along with the corresponding fluorescence lifetime values (averaged over 6 to 7.5 min) after stimulation (red). The data from (d) and (e) are used in (f) and (g), respectively. The data are also presented as mean ± SD on the right. Asterisks denote statistical significance (*t* test; $p < 0.001$).

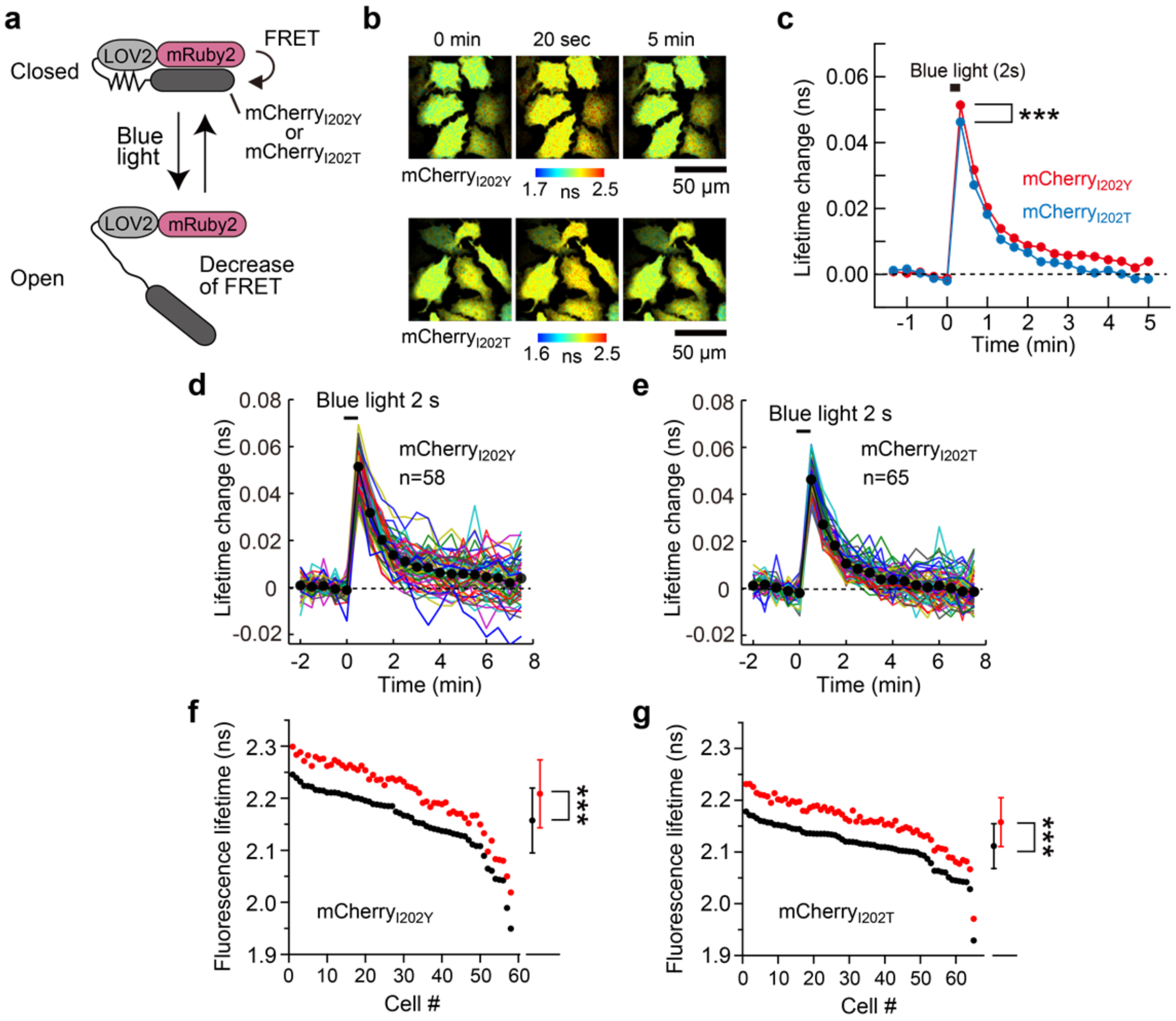

Figure S2. Performance of mCherry mutants in a LOV2 FRET sensor.

(a) A schematic of a conformational change in the light-sensitive LOV2 domain.

(b) Representative fluorescence lifetime images of the LOV2 FRET sensor in HeLa cells after illumination with a blue LED. Two-photon excitation at 1000 nm was used for excitation of mRuby2. The scale bar is 50 μm.

(c) Fluorescence lifetime changes in response to illumination with blue light. The number of cells analyzed is 58 for $mCherry_{I202Y}$ and 65 for $mCherry_{I202T}$. The data are presented as mean ± SEM. Asterisks denote statistical significance (*t* test; $p < 0.001$).

(d, e) Fluorescence lifetime changes of LOV2 FRET sensors in individual cells after illumination with blue light (the same dataset as in panel c). Colored lines represent the

response signals from individual cells, and the black circles indicate an averaged time course. The data are presented as mean ± SEM.

(f, g) The basal fluorescence lifetime (averaged over -1.3 to 0 min) of individual cells is plotted in the descending order (black) along with the corresponding fluorescence lifetime values (at 20 s) after illumination with blue light (red points). The data from (d) and (e) are used in (f) and (g), respectively. The data are also presented as mean ± SD on the right. Asterisks denote statistical significance (*t* test; $p < 0.001$).

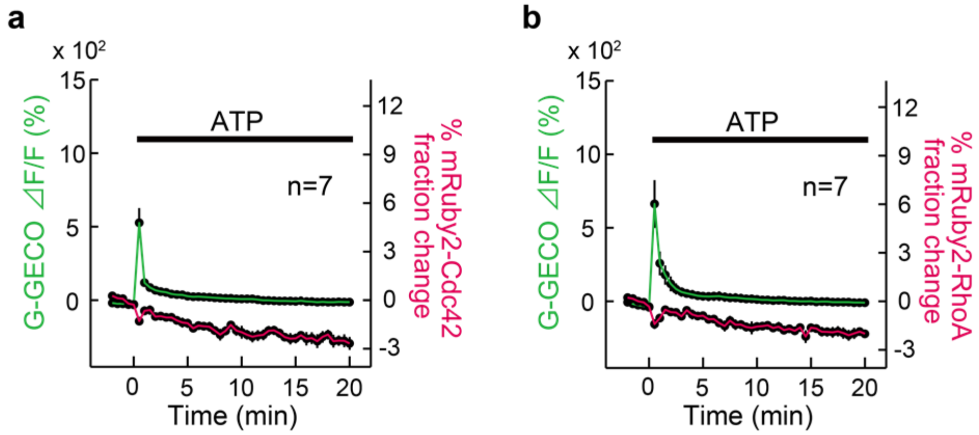

Figure S3. Negative control experiments of FLIM-FRET measurement in astrocytes. G-GECO and Cdc42 (a) or RhoA (b) FRET sensor lacking Cdc42 (mCherry$_{I202Y}$-CBD-P2A-mRuby2) or RhoA (mCherry$_{I202Y}$-RBD-P2A-mRuby2) are expressed in astrocyte, and mRuby2 binding fraction changes after 100 μM ATP stimulation were monitored.

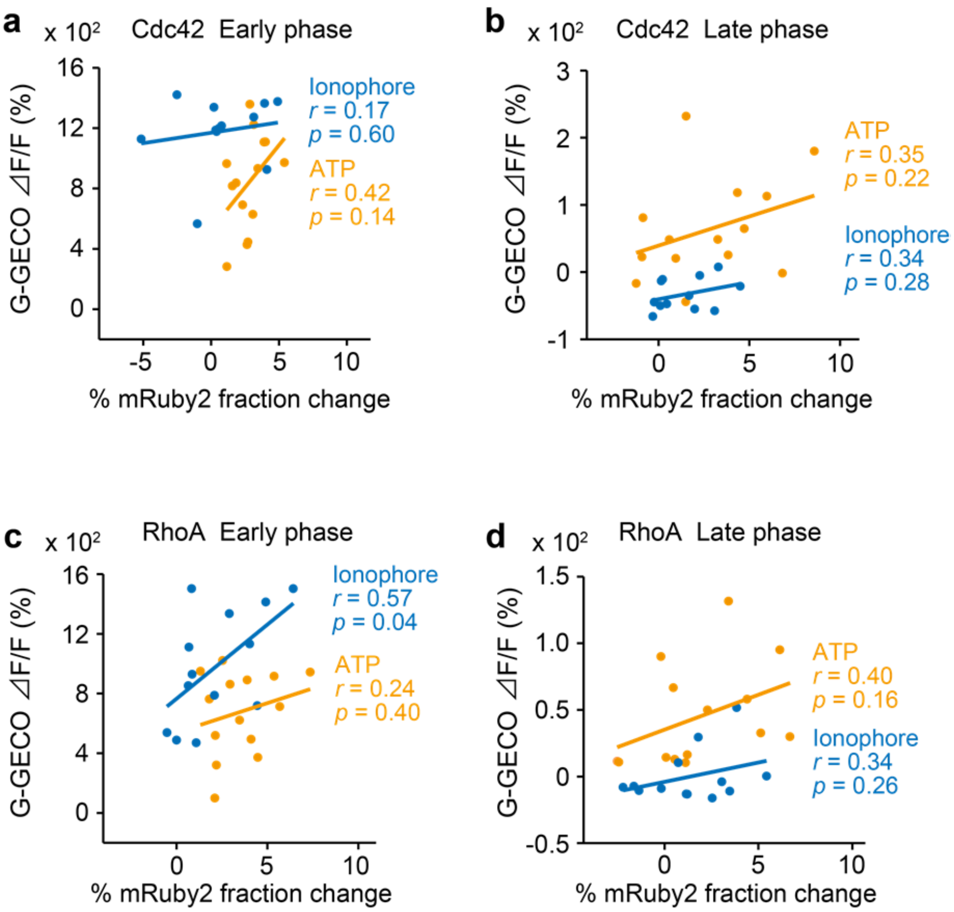

Figure S4. The correlation between the Ca^{2+} transient and Cdc42/RhoA activities in individual astrocytes.

(a, b) The correlation between the Ca^{2+} transient and Cdc42 activity (% mRuby2 fraction change) at early phase (1 min) (a) and in late phase (17–20 min) (b) after ATP (orange) or ionophore (blue) stimulation. The data set in Fig. 3c, e were used for analysis, respectively.
(c, d) The correlation between the Ca^{2+} transient and RhoA activity at early phase (1 min) (c) and in late phase (17–20 min) (d). The data set in Fig. 4c, e were used for analysis, respectively. The correlation coefficients (r) and p-values (p) are indicated in the figures.
